# Supplementary material for: Vitamin D Receptor Antagonist MeTC7 Inhibits PD-L1
Source: Cancers (Basel). 2023 Jun 30;15(13):3432. doi: 10.3390/cancers15133432 (PMC10340436; doi:10.3390/cancers15133432)
Supplement: Supplementary file 1 [file cancers-15-03432-s001.zip › cancers-2471999-supplementary.pdf]

Supplementary Materials

Vitamin-D receptor antagonist MeTC7 inhibits PD-L1

Negar Khazan[1], Emily R. Quarato[2], Niloy A. Singh[1], Cameron W. A. Snyder[1], Taylor Moore[1], John P. Miller[1,3], Masato Yasui[4], Yuki Teramoto[4], Takuro Goto[4], Sabeeha Reshi[5], Jennifer Hong[5], Naixin Zhang[1], Diya Pandey[1], Priyanka Srivastava[1], Alexandra Morell[1], Hiroki Kawano[6], Yuki Kawano[6], Thomas Conley[6], Deepak M. Sahasrabudhe[6], Naohiro Yano[7], Hiroshi Miyamoto[4], Omar Aljitawi[6], Jane Liesveld[6], Michael W. Becker[6], Laura M. Calvi[6], Alexander S. Zhovmer[8], Erdem D. Tabdanov[9], Nikolay V. Dokholyan[10], David C. Linehan[11], Jeanne N. Hansen[12], Scott A. Gerber[13,14], Ashoke Sharon[15], Manoj K. Khera[16], Peter W. Jurutka*[5,17], Natacha Rochell[18], Kyu Kwang Kim[1], Rachael B. Rowswell-Turner[1], Rakesh K. Singh*[1], Richard G. Moore[1]

Affiliations:

1. Wilmot Cancer Institute and Division of Gynecologic Oncology, Department of Obstetrics and Gynecology, University of Rochester Medical Center, Rochester, NY, USA.
2. Department of Environmental Medicine, University of Rochester Medical Center, Rochester, NY, USA.
3. Department of Microbiology and Immunology, University of Rochester Medical Center, Rochester, NY, USA.
4. Department of Pathology and Laboratory Medicine, University of Rochester Medical Center, Rochester, NY, USA.
5. University of Arizona College of Medicine, Phoenix, AZ, USA.
6. Department of Medicine, Hematology/Oncology, University of Rochester Medical Center, Rochester, NY, USA.
7. Division of Surgical Research, Rhode Island hospital, Brown University, Providence, RI, USA.
8. Center for Biologics Evaluation and Research, U.S. Food and Drug Administration, Silver Spring, MD, USA.
9. CytoMechanobiology Laboratory, Department of Pharmacology, Penn State College of Medicine, Pennsylvania State University, Hershey, PA, USA.
10. Department of Pharmacology; Department of Biochemistry & Molecular Biology, and the Center for Translational Systems Research, Penn State College of Medicine, Pennsylvania State University, Hershey, PA, USA.
11. Division of Surgery, University of Rochester Medical Center, Rochester, NY, USA.
12. Division of Neurosciences. Colgate University, Hamilton, NY, USA.
13. Division of Surgery and Microbiology and Immunology, University of Rochester Medical Center, Rochester, NY, USA.
14. Department of Radiation Oncology, University of Rochester Medical Center, Rochester, NY, USA.
15. Birla Institute of Technology, Mesra, Ranchi, India.
16. Presude Lifesciences, Delhi, India.
17. School of Mathematical and Natural Sciences, Arizona State University, Health Futures Center, Phoenix, AZ, USA.
18. Institute of Genetics and of Molecular and Cellular Biology, Cedex, France.

* Correspondence: (Primary Contact): RKS; E-mail: rakesh_singh@urmc.rochester.edu; Tel: 585-276-6281; PWJ; Email: Peter.Jurutka@asu.edu; Tel: 602-543-6087.

Cell Viability assay: Effects of MeTC7 on the cell viability were determined by MTS assay. Briefly, 25,000 cells/well (MOLM13, MV411, THP-1 and U937) or 78,000 cells (primary/untreated and relapsed AML) seeded in triplicate wells of a 96 well plate were treated with indicated doses of MeTC7 and incubated for 96 hours. Cell Titre96R Aqueous One Solution Cell Proliferation Assay reagent (Promega, catalog number: G3580) diluted 1:10 in complete RPMI medium was added and cells were incubated for additional 4-8 hours. The optical density values were read at 490 nM wavelength using BioRad iMark microplate reader.

Colony forming unit assay: To evaluate the effect of MeTC7 treatment on the clonogenic potential AML (at a concentration of 2,000 cells/ml), THP-1 (500 cells/ml), and U937 (200 cells/ml) cells were added to MethoCult H4435 Enriched (Stem Cell Technologies, cat#04435). Colonies were counted between 7 and 21 days. To evaluate the effect of MeTC7 on colony formation by normal bone marrow vials of cryopreserved aliquots were thawed in a 37^0C water bath. Nine ml of RPMI with 2% FBS were added dropwise to the cells in 1 ml of freezing media and cells were centrifuged at 1000 rpm for 5 min. cells were re-suspended the cells in 5 ml of RPMI medium. The cells were counted using a hemocytometer with viability estimated by Trypan blue dye exclusion. The cells were added to MethoCult H4435 (Stem Cell Technologies, cat#04435) to achieve concentrations of $5x10^4$ cells/ml to $1x10^5$. The mixture was vortexed and allowed to settle. The plates were incubated and colonies were counted at 7, 14, and 21 days. Images of colony forming unit assays were acquired using the Celigo imaging cytometer (Nexcelom Biosciences) at a resolution of 1 pixel/um. Celligo instrument software was used for image processing.

To evaluate the effect of MeTC7 on clonogenic potential of primary AML cells, vials of cryopreserved aliquots were thawed rapidly in a 37˚C water bath. The contents were transferred to a 15 ml conical tube, to which was added one tenth the volume of DNase (bovine pancreas deoxyribonuclease, Sigma Aldrich, cat# D4263-5VL), mixed the contents gently and incubated in a 37˚C water bath for 90 seconds. Added 9 ml of DMEM with 2% FBS dropwise and cells were mixed gently and centrifuged for 5 minutes. Cells were aspirated and discarded the supernatant. Added 70 µl of DNase and mixed gently to the cells. Added 10 ml of DMEM with 2% FBS slowly to resuspend and centrifuge for 5 to 6 minutes. Discarded the supernatant, 50 µl of Dnase was added, mixed gently and pellets were resuspended in 5 ml of DMEM + 10% FBS, L-glutamine 2 mM (GIBCO) and penicillin and streptomycin (50 units and 50 mcg/ml). The cells were counted in a hemocytometer with viability estimated by Trypan blue dye exclusion. Added cell suspension to MethoCult achieve final concentration between $5x10^4$ to $2x10^5$/ml. The mixture was vortexed and allowed to settle. Each condition was dispersed in quadruplicate at the volume of 0.5 ml/well of a four-well plate. The plates were incubated and colonies were counted at 7 and 14 days.

siRNA knockdown and stable overexpression of VDR: VDR expression in MOLM13 and THP-1 cells was knocked down using Silencer VDR siRNA (ThermoFisher, #138720) or non-targeting control using Lipofectamine 3000 (Thermo Fisher Scientific, # L3000015) following the manufacturer's protocol.

Chromatin Immunoprecipitation (ChIP): ChIP assay was performed using the Magna ChIP kit (Millipore; #17-10085) according to the manufacturer’s instructions with minor modifications. The cells were cross-linked with formaldehyde for 10 minutes at room temperature. Cell sonication

was done in nuclear buffer (four 30-s pulses, output 3.0, duty cycle 30% in ice with 120-s rest between pulses; Branson Sonifier 450). A fraction of the mixture of protein-DNA complex was used as “input DNA.” Soluble chromatin was immunoprecipitated with anti-VDRE antibody (Santa Cruz Biotechnology, #sc-13133) and normal mouse immunoglobulin G (IgG) (Santa Cruz Biotechnology, #sc-2025) directly conjugated with Magnetic Protein A beads. Immuno-precipitated DNA was eluted and reverse cross-linked, and then DNA was extracted and purified using a spin filter column. DNA samples were analyzed by PCR. PCR products were electrophoresed on 2% agarose gel, and ethidium bromide-stained DNA was visualized by a Gel Doc XR+ (BioRad, Hercules, CA).

Co-culture of mesenchymal stem cells (MSCs) with primary AML cells: MSCs isolated from the low-density bone marrow donated by consented AML patients and healthy donors following institutional protocol were grown in the vented T75 flasks as described recently. MSCs were cultured in MEMα medium (Gibco, #12561) supplemented with 10% heat inactivated FBS and 1% pen-strep antibiotics. Cultured MSCs were trypsinized (0.25%) and centrifuged at 12000 rpm for 5 minutes. 500 MSCs/well were seeded in an 8 well-slide Millicell® EZ Slides (Sigma Millipore, #PEZGS0816) overnight to adhere in complete DMEMα medium. Leukapheresis product was processed by ficoll-hypaque separation. Following a wash with PBS, isolated cells were aliquoted and cryogenically stored for use in experiments. Cells were thawed and suspended in warm MEMα without phenol red (Gibco, #41061029), 60 µg/mL penicillin and 100 µg/mL streptomycin (Gibco, #15140122). Cells were centrifuged at 120xg for 10 minutes to remove dead cells. The supernatant was aspirated and cells were resuspended at 1.0 x 10^6 cells/mL in MEMα containing 5 µM CellTracker Green (Thermo, #C7025). Cells were incubated at 37°C and 5% CO_2 for 30min. Following incubation cells were washed twice in MEMα +10% FBS (Hyclone, #SH30398.03). Cells (1.0x10^6/mL) were resuspended at in MEMα +10% FBS and 1% Pen/Strep. 50uL of this suspension was added to each chamber of a chamber slide (Millipore, #PEZGS0816). Control (DMSO) and MeTC7 treatment occurred by addition of media containing treatment into an equal volume of co-culture containing media. At 24 and 48 hours after treatment, cells were imaged using an Olympus CKX-41 inverted microscope. Image acquisition used Olympus cellSens imaging software version 2.3. Background subtracted fluorescence images were merged with brightfield images using Adobe Photoshop, release 23.2.2

Supplementary Information-S1: List and catalog numbers of the antibodies used for the flow cytometric analysis the tumors or cells.

1. CD45-FITC, BD Bioscience, cat#553080
2. CD8-PerCP-CyTM5.5, BD Bioscience, cat#551162
3. CD69-APC, BD Bioscience, cat#560689
4. PD1-PE-Cy7, Biolegend, cat#109110
5. PDL1-BV605, Biolegend, cat#124321

Abbreviations:

1. CDs: Cell differentiation markers (such as CD3, -8, -45 and -69)
2. irAEs: Immune related adverse reactions

3. MeTC7: Name of the drug candidate
4. RT: Radiation Therapy
5. PD-L1: Programmed death receptor ligand
6. PD-1: Programmed death receptor
7. VDR: Vitamin-D receptor

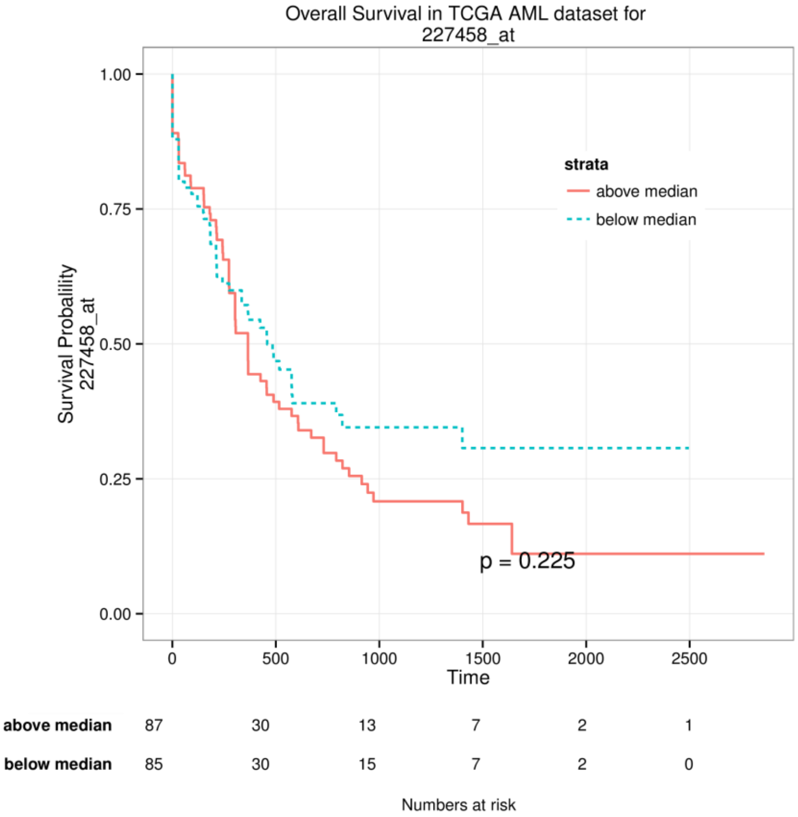

Figure-S1. **PD-L1 overexpression indicates increased mortalities among the AML patients**. Although not statistically significant, survival analysis showed increased incidences of deaths among AML patients upon increased PD-L1mRMA expression. PD-L1 mRNA expression among AML patients was analyzed using the data and inbuilt tools available at Bloodspot database (TCGA database: 227458_at).

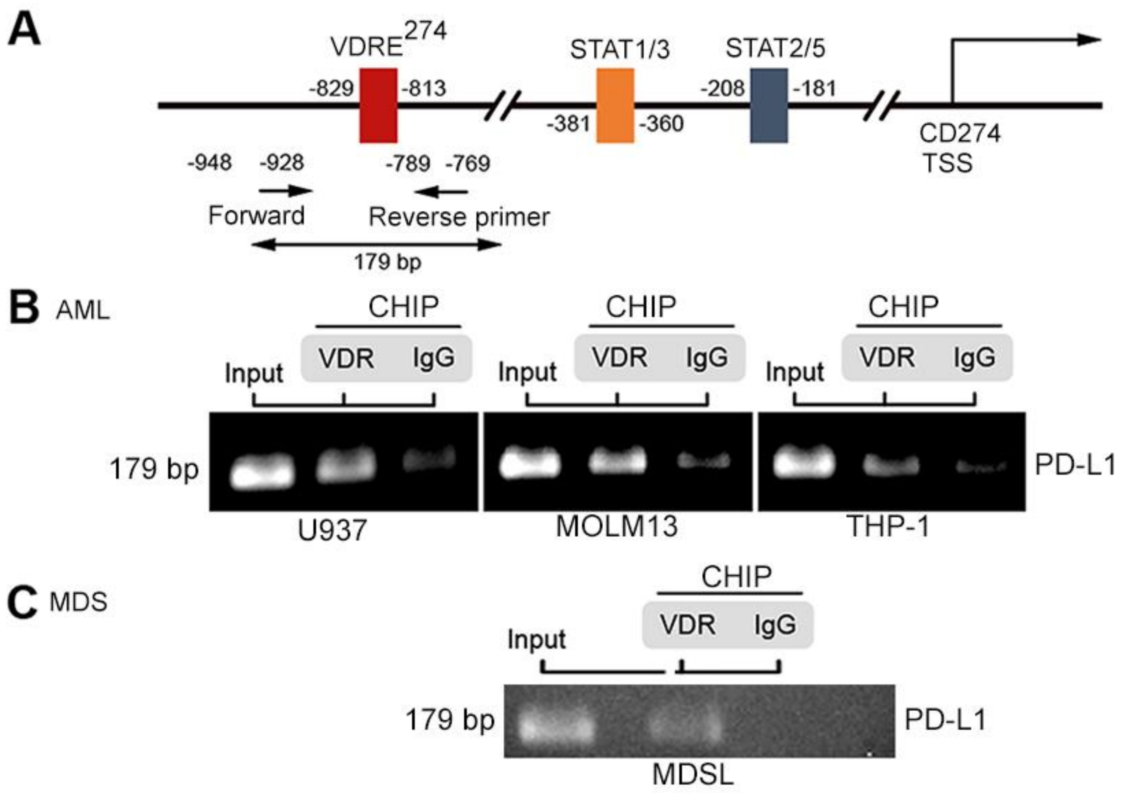

Figure-S2. Vitamin-DR/VDR transcriptionally regulate PD-L1 in AML and MDS cells. (**A**) Schema of PD-L1 promoter depicts the presence of VDRE and STAT1/3 and STAT2/5. ChIP assay in AML (**B**) and MDS (**C**) cells using a monoclonal VDR antibody captured VDRE sequence in PD-L1 promoter. Monoclonal VDR antibody (Santa Cruz Biotechnology, D6, #: SC-13133) captured VDRE sequence in PD-L1 promoter. VDRE (forward) primer sequence: 5' GGAAAGGCAAACAACGAAGA-3'; VDRE (reverse): 5'-GCGCTGAACTTCTAGGTGCT-3'.

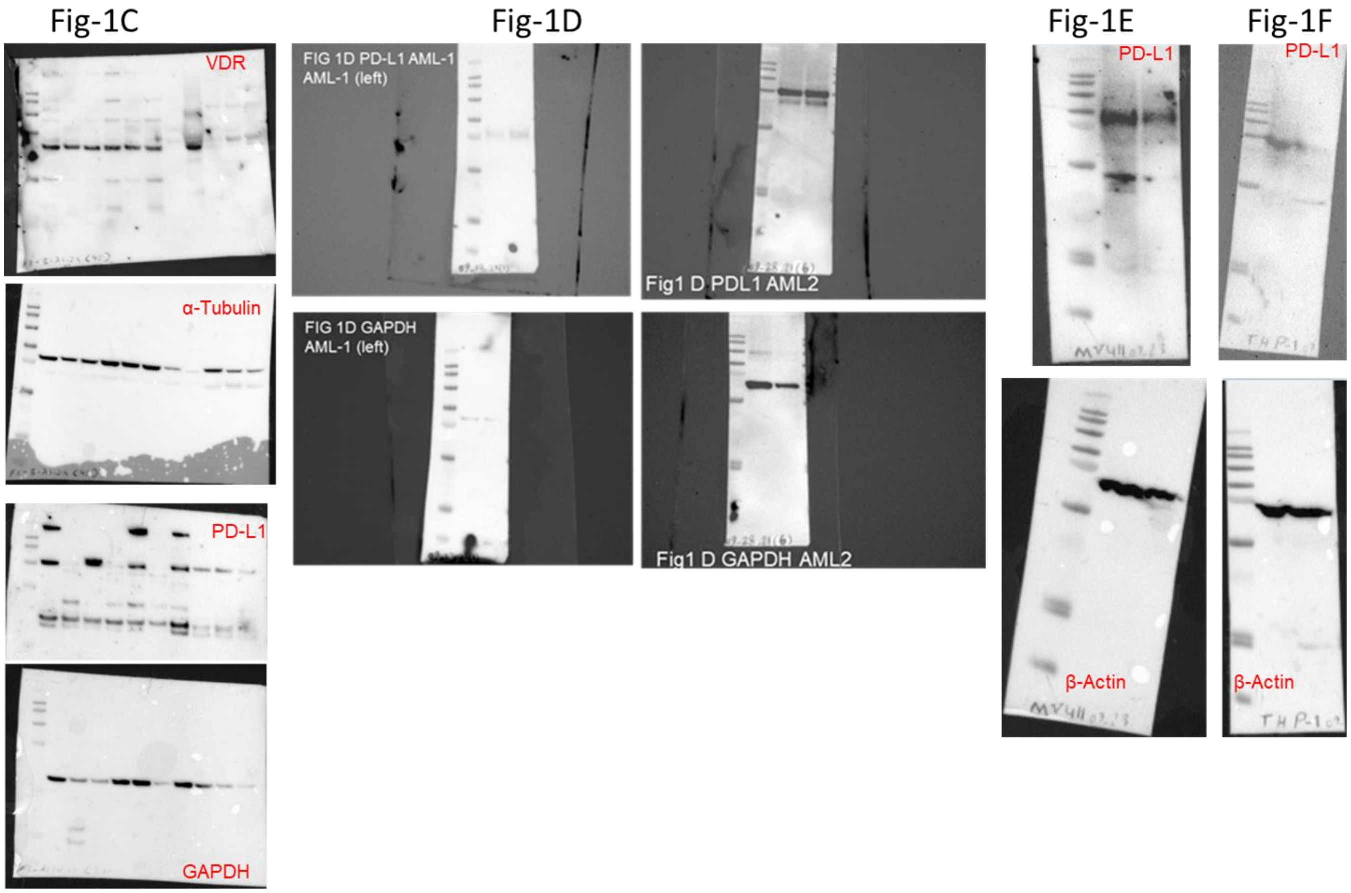

Figure-S3. Original and unaltered full scans of western blots presented in Figure-1:C-E.

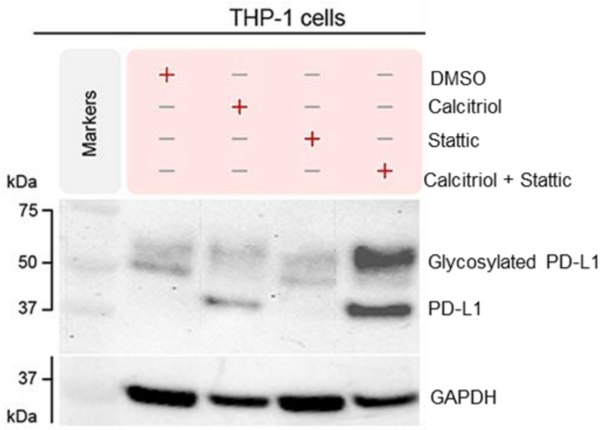

Figure-S4. **Role of STAT3 signaling in AML cell-lines.** Western blot for PD-L1 in THP1 cells treated with Stattic (0.5 µM) and/or with calcitriol (0.4 µg/mL), as indicated. Both glycosylated and non-glycosylated PD-L1 forms were detected. Membranes were stripped and re-probed for GAPDH (Cell Signaling Technology, #: 2118, dilution: 1/16,000).

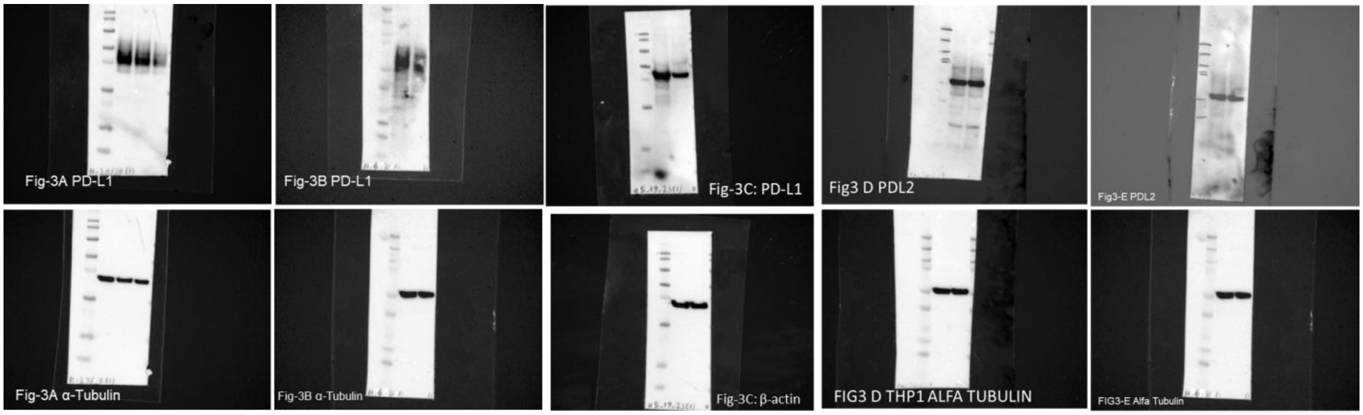

Figure-S5. Original and unaltered full scans of the western blots presented in the Fig-3: A-E.

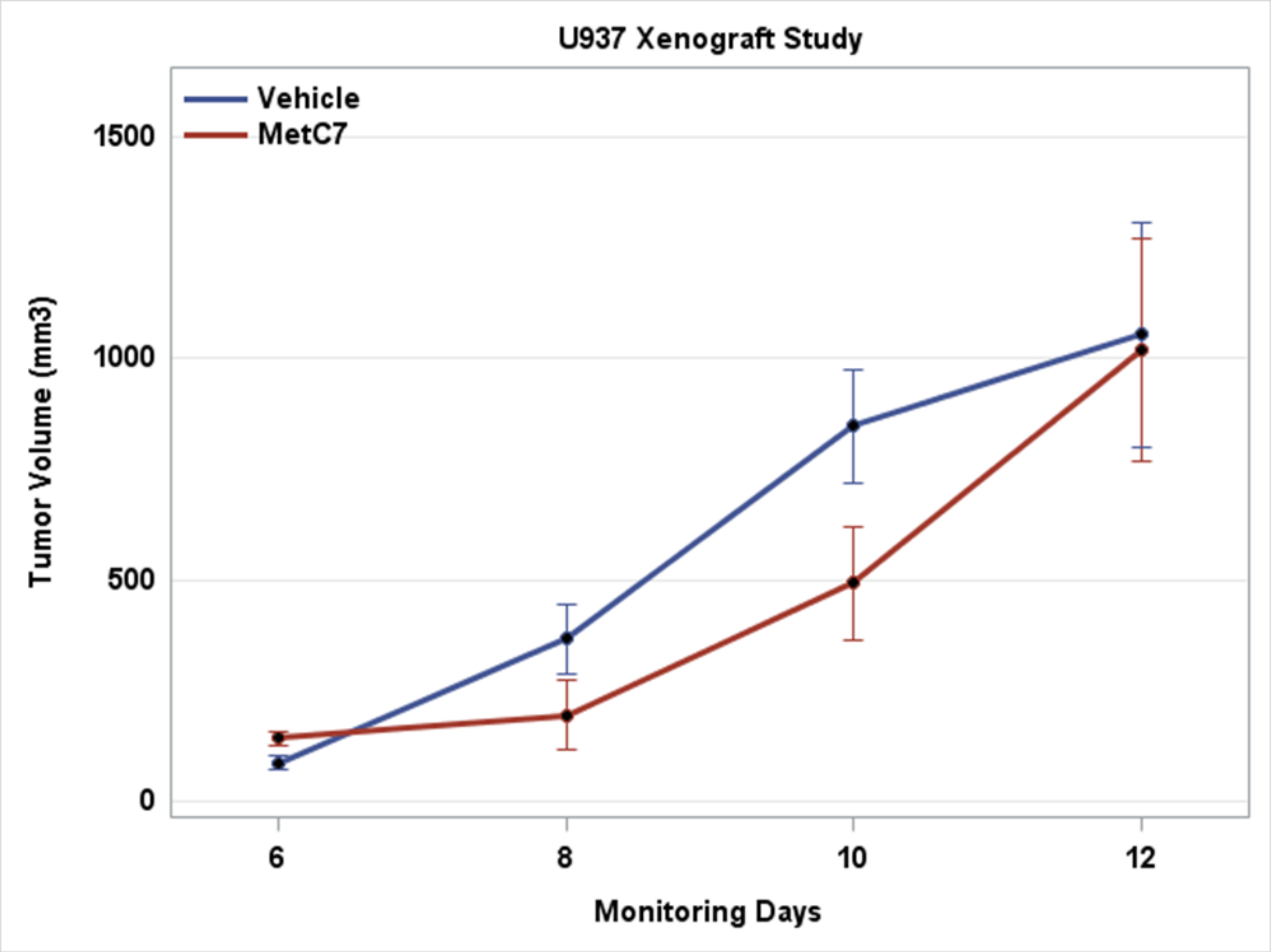

Figure-S6. The response of MeTC7 against U937 AML cell derived xenografts growing in NSG mice.
